# Evaluation of Integrated Child Health Days as a Catch-Up Strategy for Immunization in Three Districts in Uganda

**DOI:** 10.3390/vaccines12121353

**Published:** 2024-11-29

**Authors:** Mansoor Farahani, Tonny Tindyebwa, Nandita Sugandhi, Kirsten Ward, Youngjoo Park, Pamela Bakkabulindi, Shibani Kulkarni, Aaron Wallace, Samuel Biraro, Yvette Wibabara, Hannah Chung, Giles A. Reid, Driwale Alfred, Rita Atugonza, Elaine J. Abrams, Ledor S. Igboh

**Affiliations:** 1 ICAP, Columbia University, New York, NY 10032, USAgr2580@cumc.columbia.edu (G.A.R.); eja1@cumc.columbia.edu (E.J.A.); 2 ICAP at Columbia, Kampala, Uganda; 3 U.S. Centers for Disease Control and Prevention, Global Immunization Division, Global Health Center, Atlanta, GA 30329, USA; 4 U.S. Centers for Disease Control and Prevention, 1577 Ggaba Road, Kampala, Uganda; ssu4@cdc.gov; 5 Uganda National Expanded Programme on Immunization, Ministry of Health, Lourdel Road, Kampala, Uganda

**Keywords:** Integrated Child Health Days (ICHDs), immunization coverage, vaccine uptake, catch-up vaccination strategy, EPI, Uganda

## Abstract

**Background:** Uganda’s Integrated Child Health Day (ICHD) initiative aims to improve children’s access to vaccinations. Although widely used as a catch-up vaccination strategy, the effectiveness of the ICHD program in increasing immunization coverage, especially among vulnerable populations, has not been recently evaluated. This study assessed the reach and uptake of ICHD for immunizations in Uganda. **Methods:** A mixed-methods evaluation was conducted in three districts (Rakai, Kayunga, and Bukedea) where ICHDs occurred. The data collection included a cross-sectional household survey using validated WHO-adapted questionnaires of 1432 caregivers of children under five years old, key informant interviews with 42 health managers and workers, and nine focus group discussions with caregivers between October and December 2022. The vaccines assessed were Bacillus Calmette–Guerin, oral polio, Pentavalent, pneumococcal conjugate, rotavirus (RV), and measles-rubella (MR). **Results:** The immunization coverage based on child health cards was over 90% for all vaccines except for the second dose of RV (88.3%) and MR (16.2%). Among the children, 2.3% had received no Pentavalent vaccine, and 69.4% were fully vaccinated for their age. Of the 631 children who attended ICHDs, 79.4% received at least one vaccine during the event. Village Health Teams (49%), health workers (18.3%), and megaphone outreach (17.9%) were the primary information sources. Key informants cited challenges with coordination, vaccine delivery, and mobilization. **Conclusions:** Despite operational challenges, ICHDs appear to have contributed to routine childhood vaccinations. Further research is needed to assess the sustainability and cost-effectiveness of the program.

## 1. Introduction

At the 2020 World Health Assembly, all 194 member states, including those in the African Region, endorsed the Immunization Agenda 2030 (IA2030), the 2021–2030 global strategy that envisions a world where everyone, everywhere, at every age fully benefits from vaccines [1]. In many sub-Saharan African countries, routine immunization as a primary healthcare service requires additional resources to achieve universal coverage [2]. Therefore, it is necessary to intensify critical routine immunization services to reduce immunity gaps periodically [3]. Integrated Child Health Days (ICHDs), also known as Child Days Plus, are a form of periodic intensification of routine immunization (PIRI) [4]. They are intended to boost routine immunization coverage by improving access to vaccination and increasing the frequency and scope of immunization services in the community [5]. In Uganda, ICHDs are implemented as biannual (April and October) month-long, country-wide initiatives that deliver a comprehensive package of child survival interventions. Uganda has an estimated 16% rate of un- or under-vaccinated age-eligible children. ICHDs were considered to help address localized areas of low vaccination coverage, provide additional child health services, promote equity, prevent disease outbreaks, and strengthen systemic factors within the healthcare system [6].

During the 1960s and 1970s, Uganda achieved high vaccination coverage against polio and tuberculosis (with the Bacillus Calmette–Guerin (BCG) vaccine). However, political unrest in the 1980s contributed to a drop in BCG coverage [7]. In response, the Government of Uganda launched the Uganda National Expanded Program on Immunization (UNEPI) in 1983. The UNEPI’s implementation led to notable improvements in vaccination coverage rates nationwide. It expanded the age range of vaccines, introduced new vaccines, and increased access to immunization services, especially in remote and underserved areas [8]. Government funding cuts in the 1990s affected immunization services in Uganda, and decentralization left districts with insufficient resources for management and financing, resulting in a decline in routine vaccination coverage [9]. In Uganda, the ICHDs were introduced in 2004 and have since included service packages of vaccines, vitamin A supplementation, deworming, growth monitoring, and, on some occasions, insecticide-treated bed net distribution [10].

As of 2019, Uganda, through UNEPI, had protected children against a variety of vaccine-preventable diseases using the following vaccines: BCG; oral polio vaccine (OPV); diphtheria–tetanus–pertussis–hepatitis B–*Haemophilus influenza* type b (Pentavalent, hereafter Penta); Pneumococcal Conjugate Vaccine (PCV); rotavirus vaccine (RV)); and measles-rubella vaccine (MR) [11].

In Uganda, the ICHDs were introduced in 2004 as part of the country’s national strategy to strengthen routine immunization services. This initiative aligned with WHO’s global strategy for PIRI, which encourages combination approaches to reach everyone targeted for immunization. The program was implemented by the Ministry of Health (MoH) through the UNEPI. ICHDs in Uganda provide a comprehensive service package including the following:

- Routine childhood vaccinations according to the national schedule.
- Vitamin A supplementation.
- Deworming.
- Growth monitoring.
- Nutritional counseling.
- Family planning services.
- Health education.

During the October 2022 ICHD, additional services included COVID-19 awareness and, in select districts, hepatitis B vaccination for those over 18 years.

In Uganda, vaccination services are provided by the MoH through the UNEPI at national, regional, district, and community levels, with other stakeholders involved at each level [12]. In 2019, Uganda’s national vaccination coverage was above the global target of 90% for the third dose of the Penta vaccine (93%), OPV (92%), and BCG (94%), with coverage above 85% for RV (87%) [13]. However, the global COVID-19 pandemic reduced the availability of primary healthcare services, including vaccination, thus hampering progress toward achieving the targets set in Uganda’s comprehensive Multi-Year Strategic Plan for immunizations 2016–2020 [14].

Despite many years of intensifying routine immunization through ICHDs and using it as a strategy for catch-up vaccination for children < 12 months of age in Uganda, the implementation and effects of this strategy on the uptake of a wide range of vaccines have not been evaluated since 2009 [15]. This evaluation aimed to understand the effectiveness of the ICHD program in Uganda in reaching unimmunized and under-immunized children and suggest recommendations for service delivery improvements in three districts. Furthermore, we documented the key components of the ICHD program implementation and perspectives from health managers and healthcare workers, assessed caregivers’ motivators and barriers to ICHD attendance, and estimated ICHD attendance among children < 5 years of age.

## 2. Materials and Methods

A mixed-methods evaluation was conducted in three districts between October and December 2022. We used national administrative immunization coverage data for Penta 3 and MR, the number of un- and under-vaccinated children, and the Reach Every District categorization to select the districts (The Reach Every District categorization is a guide and strategy to achieve 80% immunization coverage in all districts and 90% nationally in WHO member African states. 2017, World Health Organization). Final decisions on the districts included in the evaluation depended on the additional operational context of the national immunization program as guided by UNEPI. The selected districts were Bukedea (high-performing or >90% coverage), Kayunga (medium-performing or >80% coverage), and Rakai (poor-performing or <80% coverage) in Uganda’s Eastern, Central, and South-Western regions, respectively. In the selected districts, data were collected through household surveys, key informant interviews (KIIs), and focus group discussions (FGDs). Prior to implementation, all data collection tools were piloted in similar, non-selected settings, resulting in minor format and content modifications.

### 2.1. Data Collection

#### 2.1.1. Household Survey Procedures

We applied two-stage cluster sampling [16]. Assuming an immunization coverage rate for the Integrated Child Health Days of 85% and allowing for a 10% non-response rate, we calculated that we would need 371 households and 25 EAs per strata to achieve a 10% precision level and a 90% confidence interval in each stratum. In the first stage, using the available data from the 2014 census [17], we randomly selected 75 enumeration areas proportional to each district’s total population size. We carried out HH listing and collected data on the number of HH members, the sex and age of children, and their primary caregivers. In the second stage, we randomly selected 15 HHs from each enumeration area among those initially listed with eligible children < 5 years of age. If a household had more than one child under 5 years of age, up to five children were randomly selected using a random number generator, and data collection focused on one selected child at a time. All the primary caregivers who consented were enrolled.

Using a structured questionnaire adapted from WHO’s standardized household survey tools for immunization coverage assessment [18] we interviewed the primary caregiver in each selected HH. The questionnaire underwent a rigorous validation process including an expert review for face and content validity by immunization specialists from UNEPI, CDC, and ICAP; translation and back-translation to ensure linguistic equivalence in local languages (Luganda and Itesot); and pilot testing in similar, no n-selected settings. The pilot testing assessed intelligibility, consistency, and cultural appropriateness, leading to the refinement of the final instrument. The validated questionnaire collected data on the caregiver and child’s socio-demographics, child vaccination history from both caregiver recollections and a review of the child’s Child Health Card, caregiver knowledge, attitudes, and practices related to childhood immunization and uptake of ICHD services, which are offered twice a year. The study team, the US CDC, the Uganda National Council for Science and Technology, and the Uganda Virus Research Institute reviewed and approved them.

#### 2.1.2. Key Informant Interviews (KII) Procedures

We conducted KIIs with purposely selected key health officials involved in ICHD planning and implementation at the national (N = 9), district (N = 12), and health facility levels (N = 21) to understand the health system perspectives of conducting ICHDs and related barriers and enablers. Health officials at the national and district levels are involved in planning the ICHDs; Village Health Teams coordinate with district officials to plan and implement ICHD activities, and VHTs support social mobilization efforts for ICHDs (Village Health Teams in Uganda comprised of four-member Community Health Worker teams provide home visits and health management services for local communities. Each team has at least two members who provide integrated community case management of childhood illnesses).

We interviewed 42 key informants from the health and administrative structure of the UNEPI and the MOH at the national, district, and health facility levels, including 9 national EPI Program officers from the government and international organizations, 6 district officers (the District Health Officer and Assistant District Health Officer in each district), and 21 healthcare workers (HCWs) at the hospital, health center level IV, III, and II, and outreach posts involved in delivering routine vaccines (Uganda’s health facilities are classified into seven levels based on the services they provide and the area they intend to serve. The facilities are designated as Health Centre Level One (HC I) to Health Centre Level Four (HC IV): general hospital, Regional Referral hospital, and National Referral hospital). Respondents who had a supervisory role overseeing routine immunization service delivery for at least 12 months were purposively selected to represent each level of the ICHD’s service delivery cascade at health centers. Interviews were set up at the participants’ workplace at a time that suited their schedules.

The interviewers used a pre-designed interview guide to elicit information on immunization service delivery, management, finance, data and surveillance, cold chain and vaccine management, advocacy, communication, social mobilization, and challenges and recommendations. The interviews were conducted in English or Luganda and recorded on tablets with KII guides installed. Trained research assistants translated and transcribed each interview recording verbatim.

#### 2.1.3. Focus Group Discussion (FGD) Procedure

Three FGDs were conducted in each district (a total of nine FGDs) with caregivers of children < 5 years of age to understand their knowledge, attitudes, and practices related to ICHDs. The VHTs helped identify caregivers who had or had not attended ICHDs and helped recruit and select FGD participants. Each FGD comprised eight caregivers of children 6–59 months old, with a mix of those whose children were incompletely vaccinated and those who had received all nationally recommended vaccines for their age. FGD participants were mobilized in central meeting venues such as churches, community halls, and school compounds within their community’s radius. Each discussion lasted between 60 and 90 min.

Each FGD was conducted in the district’s local language, facilitated by a trained facilitator, accompanied by a note-taker, and recorded to document the discussion. After each discussion, a debriefing session was conducted between the facilitator and note-taker. A debriefing form was completed to identify and report any key discussion themes. Verbal informed consent was obtained from all participants and documented electronically by trained data collection staff.

The interview guides for KIIs and FGDs are provided as Supplementary Materials. They cover topics such as program implementation, challenges, and recommendations across the five pillars of immunization strategy.

### 2.2. Data Analysis

#### 2.2.1. Household Survey (Quantitative Analysis)

The household data were cleaned to remove duplicates and improbable values and weighted based on sampling strategy and clustering to ensure representativeness. We conducted descriptive analyses, including frequencies, means, and proportions, and reported related 95% confidence intervals for the estimates. Analyses were performed in SAS^®^ 9.4 M8 (SAS Institute, Cary, NC, USA). The child’s vaccination status is based on data from the receipt of specific vaccines through data abstracted from vaccination cards.

Vaccination completion was based on the Uganda Immunization Schedule (Appendix A). In our analysis of vaccine coverage, we targeted children aged 9–59 months to evaluate the completion of the vaccine series starting from early infancy through the second year of life, based on the Uganda National Immunization Program schedule. This age range was chosen because it captures both the initial and booster doses of essential vaccines such as DTP, Polio, Hib, and measles, allowing for a comprehensive assessment of vaccination coverage and its effectiveness in preventing diseases during a crucial developmental period. We defined a child’s vaccination status as delayed if the 1st dose of Penta, OPV, PCV, or RV was received >8 weeks of age; the 2nd dose of Pentavalent, OPV, PCV, or Rota was received >4 weeks after the first dose; the 3rd dose of Pentavalent, OPV, or PCV was received >4 weeks after the 2nd dose; the 1st MR dose was received >10 months; or the 2nd MR dose was received >18 months. Any child who missed one or more of these vaccine doses was considered incompletely vaccinated for their age. We defined zero-dose as a child without any Penta vaccine doses as defined by the Uganda EPI, Ministry of Health [19]. We also assessed the number of children who received age-eligible vaccines during the October 2022 ICHDs based on a review of the Child Health Cards. Other variables related to ICHD knowledge and uptake were reported as recorded from the questionnaire.

#### 2.2.2. KIIs and FGDs (Qualitative Analysis)

We analyzed the qualitative data by reading and coding all the transcripts to identify key themes. First, five coders coded three transcripts to gather consensus on the coding, an interactive process of reviewing, discussing, and refining codes. Second, a final codebook was developed based on deductive and inductive coding to code all transcripts. Finally, the codes were further categorized into groups to identify key themes related to barriers and facilitators of implementing ICHDs and related uptake and recommendations for improving ICHD services. We documented these themes as key outcomes of our evaluation and included relevant quotes to support our findings. Illustrative and anonymized quotes from participants provided their voices on the different themes.

The Child Health Card is a critical document in Uganda’s healthcare system, serving as the primary record of a child’s vaccination history. It is typically issued at birth in health facilities or during a child’s first contact with health services. Although 79.9% of children in this study were born in health facilities, only 67.1% had Child Health Cards at the time of the survey. This gap may be due to cards being lost, damaged, or never issued.

Analyses requiring precise vaccination dates and adherence to schedules—such as coverage rates and timeliness—relied on documented evidence from Child Health Cards (n = 980). However, analyses of ICHD attendance and service utilization included all participants, regardless of card availability, to provide a comprehensive assessment of program reach. This approach balanced the need for accurate vaccination timing data with an inclusive evaluation of service access.

#### 2.2.3. Ethical Considerations

The study protocol was reviewed and approved by the U.S. CDC Global Health Center’s Office of the Associate Director for Science (CGH/IDST-6/3/22-461ec), the institutional review boards at the Mailman School of Public Health, Columbia University, Uganda Viral Research Institute, and Uganda National Council of Science and Technology. Written informed consent was obtained from all participants prior to data collection. For the household survey, FGDs, and KIIs, participants were provided with detailed information about the study objectives and procedures in their local language, and their right to withdraw at any time was emphasized.

## 3. Results

### 3.1. Characteristics of the Study Population

#### 3.1.1. Children, Caregivers, and Household Characteristics

Caregivers of 1432 children aged 0–59 months old participated in the household survey across the three districts. The largest age group of children (40.7% [37.9–43.6%) was that of children aged 0–9 months old (Table 1). The majority of the children (79.9% [95% CI: 77.8–82.9%]) were born in a health facility. Most of the respondents (76.7% [95% CI: 77.1–81.0]) were the biological mothers of these children, with an average age of 33.1 years (95% CI: 32.3–33.9). Most of the caregivers had no formal or only primary education (63.1%), lived in remote villages (74%), and subsistence farming was their primary source of income (72%).

#### 3.1.2. Child Immunization Status

Among all the participants, 980 (67.1%) children had Child Health Cards for vaccination. Among the children with a Child Health Card, 69.4% (95% CI: 66.8–72.1%) received all recommended vaccines for which they were age-eligible based on the Uganda National Immunization Program schedule. An analysis of the vaccine coverage revealed statistically significant differences across districts (*p* = 0.011), with Rakai showing the highest proportion of fully vaccinated children (76.1%), followed by Kayunga (67.2%) and Bukedea (63.5%). However, ICHD attendance rates were comparable across districts (*p* = 0.292), with approximately 46.1% attending in Bukedea, 41.1% in Kayunga, and 38.2% in Rakai.

A further analysis of the factors influencing the vaccination status revealed that the caregiver age and marital status were significantly associated with child vaccination status. A chi-square test indicated that the caregivers under 30 years of age were less likely to have fully vaccinated children compared to those aged 30 and above (*p* < 0.001). Similarly, the unmarried caregivers showed lower rates of child vaccination compared to the married caregivers (*p* < 0.001).

The vaccination coverage among the children < 5 of age across the three districts was above 90% for most of the vaccine doses except for the second dose of RV (88.3% [95% CI: 85.8–90.8%]) and MR (16.2% [95% CI: 13.5–19.0%]). We identified 33 (2.3%) children who had not received any Penta vaccine (zero-dose children), of whom 85.8% (95% CI: 72.7–98.9%) were 0–6 months old (Table 2).

Among the sampled children aged 9–59 months of age with a Child Health Card (n = 793), the coverage of the BCG vaccine, each dose of Penta vaccines, and each dose of OPV exceeded the national target of 90% (96–99%). The coverage of the first dose of PCV and Rota was at 96% and 91%, respectively. The MR coverage was 90% for the first dose but only 16% for the second dose (Table 2).

**Figure 1:**
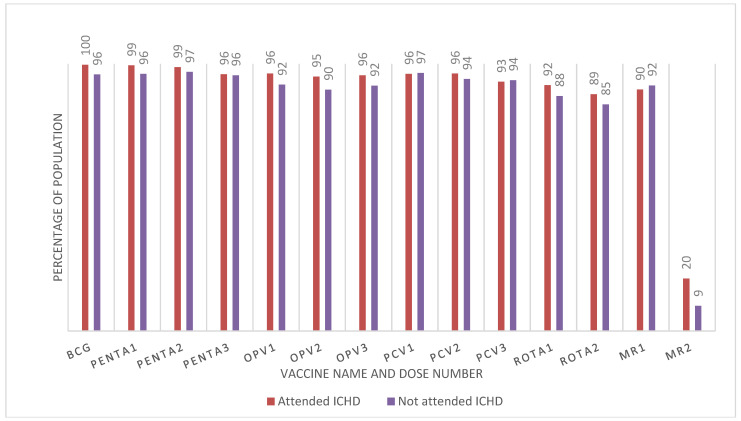
Vaccination by vaccine type and ICHD attendance among children under five years of age, Uganda, 2022 (based on household survey and Child Health Card Records). Attended 558, not attend = 111 = total 669; Bacillus Calmette–Guerin (BCG); oral polio vaccine (OPV); diphtheria–tetanus–pertussis–hepatitis B–*Haemophilus influenza* type b (Penta); Pneumococcal Conjugate Vaccine (PCV); rotavirus vaccine (Rota); measles-rubella vaccine (MR). The numbers indicate 1st, 2nd, and 3rd doses.

Figure 1 shows the vaccination rates among children < 5 of age who attended the ICHD in October 2022 and those who did not participate, based on the Child Health Card reviewed during the household visit. There was no statistically significant variation in the immunization coverage by vaccine dose between children who attended and those who did not participate in the ICHDs.

#### 3.1.3. Timeliness of Immunization

A considerable proportion of the children experienced delays in receiving routine vaccines. Similar patterns of delay were observed across multiple vaccines, with approximately half of the children experiencing delays in receiving the second and third doses. For the Penta vaccine, 38.5% (95% CI: 34.8–42.1) of the children were delayed in receiving the first dose, while delays were more common for the second (54.7% [95% CI: 51.1–58.4%]) and third doses (55.3% [95% CI: 51.5–59.0%]). Similar proportions of delay were observed with the OPV (52.4% [95% CI: 48.6–56.2%] for the second dose and 57.4% [95% CI: 53.6–61.1%] for the third dose) and PCV. For the RV, around half of the children experienced delays in both doses. The MR vaccine showed a different pattern, with about 29.6% (95% CI: 25.7–33.5%) receiving the first dose late and the second dose often being administered well beyond the recommended age range (Table 2).

### 3.2. Caregivers’ Knowledge, Attitudes, and Practices

#### 3.2.1. Caregiver Attendance of ICHDs and Related Knowledge, Attitudes, and Practices

Of the 1077 caregivers who responded to the related question, 801 (74.7% [95% CI: 71.9–77.6%]) had heard about ICHDs; of these, 423 (53% [95% CI: 53.0 (49.5–56.4]) knew the months of the ICHDs. Among the 792 participants who responded to the question about ICHD attendance, 488 (59.5% [95% CI: 55.6–63.3%]) had attended the most recent October 2022 ICHD. Among the 295 participants who reportedly did not attend the most recent ICHD, 143 (48.5%, 95% CI: 42.1–54.8) had attended previous ICHDs. The knowledge, attitudes, and practices of caregivers attending an ICHD (n = 631) are shown in Table 3. Among those who attended the ICHDs, most caregivers, 91.9% (95% CI: 89.5–94.4%), sought immunization services at the ICHD. Among the 631 (44%) children who had attended the ICHDs, most (79.4%) reported receipt of at least one vaccine during the ICHD. Regarding the motivation for attendance, one of the caregivers from a focus group was quoted as saying the following:

“*What motivates me is, as a young child, our parents did not take us for ICHDs because of misconceptions that they were immunizing the uterus so that we may not have children in the future, so for those who got the chance and were vaccinated, they didn’t get the diseases. However, all the children who were not immunized got sick. This means that when one is immunized, it prevents diseases and infections*”.

(Caregiver, FGD, Kayunga)

Eighty-five percent (95% CI: 81.7–85.5%) of the caregivers reported accessing ICHD services through service centers and outreach sites, most of whom, 85.6% (95% CI: 82.5–88.8%), traveled less than 5 km to reach them. There was no statistically significant difference between the proportion of ICHD attendees and nonattenders who traveled less than 5 km to the service site (χ^2^ test, *p* = 0.995). The most used sources of information about ICHD service availability and accessibility were VHTs (49.0% ([95% CI: 46.6–51.6%]), health workers (18.3% [95% CI: 15.5–21.1%]), and megaphone outreach (17.9% [95% CI: 17.4–20.5%). A VHT coordinator further expanded their role in communicating about the ICHDs in the community:

“*If ICHDs are going to take place on a Saturday and a Sunday, we will start the mobilization on Monday because it is a big village. We can easily find out who did not [receive the message] from the megaphone; it is where we always start before coming to those who are nearer and can gather at an outreach point. For other places where caregivers always turn up, we mobilize using the local radio. We inform them about when health workers will come for immunization.*”

(VHT, KII, Kayunga)

#### 3.2.2. Caregiver Nonattendance of ICHDs and Related Knowledge, Attitudes, and Practices

The FGDs revealed the following reasons for caregivers not attending ICHDs: inadequate social mobilization, confusing multiple ongoing immunization campaigns, doubts about vaccine safety, negative HCW attitudes, and delayed and insufficient vaccine stocks. Some who attended ICHDs voiced some concerns.

Expressing her concerns about community mobilization, one caregiver was quoted as saying the following:

“*But for me, what confuses me and the main reason I don’t immunize is because they don’t tell you which disease they are vaccinating against. But they bump into us and tell us to take the children for vaccination. However, I love vaccination, and I also took my child for routine immunizations, but not these Integrated Child Health Days without cards. Without proper sensitization, I can’t take my child for ICHDs. Even during COVID, I participated because I was informed and sensitized about it.*”

(Caregiver, FGD, Kayunga)

Regarding the HCWs’ performance at ICHDs, one of the caregivers who attended an ICHD was quoted as saying the following:

“*The other challenge is that health workers are rude (babogola), some don’t care and pay attention to the right vaccines to immunize and even sometimes end up administering the wrong dose, instead of giving a child the dose at 6 months they give them the one for 9 months.*”

(Caregiver, FGD, Rakai)

There were several immunization campaigns, such as the polio vaccine and measles, as well as the routine vaccination that occurred in the year before the ICHD, which had confused caregivers. One of them, who did not attend ICHDs, was quoted as saying the following:

“*We usually ask why, for example, they immunize every month and every year. We think if they immunize, say like, in January, then do it again next year, then it would not be confusing, but every month? So, if you are soft-hearted, you may fail to attend.*”

(Caregiver, FGD, Rakai)

### 3.3. ICHD Implementation Challenges

The KIIs of key health officials also revealed that Uganda’s ICHDs had a wide variety of challenges that hampered effectiveness and efficiency. At the national level, procedural delays in fund disbursement and accountability impeded the timely execution of activities. District operations struggled with chronic underfunding, leading to inadequate preparation and insufficient supervision, further exacerbated by simultaneous campaigns that confused staff and the public. Health facilities faced dire consequences of these financial delays, manifesting in demotivated personnel, severe staffing shortages, and logistical nightmares in accessing remote areas. Additionally, general obstacles persisted, such as a resistant public wary of vaccine safety and benefits, insufficient outreach transportation, and a shortfall in social mobilization efforts due to a lack of communication tools and funds. These systemic issues at the national, district, and facility levels, coupled with societal challenges, created a complex environment that constrained the reach and success of ICHDs.

## 4. Discussion

This evaluation of ICHDs in three Uganda districts estimated the coverage for most routine childhood vaccines to be above national targets (>90%). However, a direct association between participation in ICHDs and coverage was not found. High participation in ICHDs indicates their role in contributing to immunization coverage in the population. The evaluation found that while 3.3% of the children were zero-dose (no Penta vaccines), the ICHD program successfully provided catch-up vaccinations to 49% of the children who had missed their recommended vaccination timeline. This demonstrates the program’s effectiveness in reaching children who might otherwise have remained under-vaccinated, though continued efforts are needed to reach the remaining zero-dose children. Although attempts have been made to address vaccination delay through caregiver education and the expansion of immunization service delivery points, delays in the receipt of routine vaccines are still prevalent, particularly for vaccines in the first four months of life [20], suggesting systemic challenges that affect the timely receipt of recommended childhood vaccines in Uganda. Most of the caregivers (75%) in the three districts were aware of the ICHDs. They reported attendance in April or October 2022, mainly at outreach sites, illustrating the reach of social mobilization and communication strategies and the importance of accessibility of ICHD locations.

While the coverage for most vaccines exceeded 90%, lower coverage was found for the second dose of the rotavirus vaccine (88.3%) and the second dose of the measles-rubella vaccine (16.2%). The low MR2 coverage reflects its recent introduction to Uganda’s routine immunization schedule and logistical challenges in vaccine delivery and follow-up visits.

Similar challenges in completing multi-dose vaccine series were observed in Zambia and Somalia, particularly for newer vaccines. Strengthened follow-up mechanisms, improved vaccine stock management, and targeted communication strategies are needed to ensure the completion of these series [15,21].

Our findings contribute to a growing body of evidence on the periodic intensification of routine immunization in sub-Saharan Africa. Similarly to our observations in Uganda, evaluations of Child Health Days in other settings have demonstrated both the potential and challenges of such approaches. A multi-country assessment by Doherty et al. (2010) [5] found that while Child Health Days could rapidly improve intervention coverage, its effectiveness depended heavily on the existing health system capacity and community engagement. This aligns with our findings regarding the impact of procedural delays and community mobilization challenges on ICHD implementation.

Financial delays, insufficient social mobilization, and logistical challenges in reaching remote communities were significant barriers identified, consistent with findings from other evaluations of Child Health Days in Africa. These challenges, including chronic underfunding and difficulties accessing remote areas, indicate systemic barriers to implementing periodic intensification strategies [15].

Recent research by Arambepola et al. (2021) [21] in Zambia emphasized how vaccination campaign effectiveness varies by local context. It highlighted the critical role of targeted community engagement—findings that resonate with our observations about the importance of clear communication and community mobilization in Uganda’s ICHDs. Their emphasis on identifying and reaching zero-dose children aligns with our finding that 3.3% of the children remained unvaccinated despite the high overall coverage.

Our evaluation adds distinct value to this literature by providing a detailed examination of the ICHDs in Uganda’s specific context, particularly highlighting the complex interplay between routine immunization services and periodic intensification strategies. Unlike previous studies, our mixed-methods approach allowed us to triangulate quantitative coverage data with rich qualitative insights from both vaccination service providers and recipients, offering a more nuanced understanding of how ICHDs function within Uganda’s health system.

While the ICHDs could not reach every child, which aligns with previous literature about ICHDs from the African region [4,5], we could not find a statistically significant difference in vaccination coverage between those who attended Uganda’s ICHDs and those who did not. One possible reason for this is that the high overall coverage might limit the ability to see the effect of other contributing system-related factors. In this aspect, our findings were not consistent with prior studies about ICHDs [15,21,22], including an economic evaluation of Child Health Days in Somalia, which found that despite the high operational costs, ICHDs are a highly cost-effective service delivery strategy for addressing the major causes of child mortality in conflict-ridden areas like Somalia. In one of these studies, they compared favorably with other interventions rated as the health sector’s “best buys” in sub-Saharan Africa [22].

Our findings provide important insights into both the reach and comprehensive service delivery of ICHDs. Regarding accessibility, 74% of caregivers resided in remote villages, with 85.6% traveling less than 5 km to reach ICHD services. The program’s outreach strategy effectively served 85% of attendees through community-based sites, suggesting success in extending services beyond health facilities. However, qualitative data from health workers highlighted persistent challenges in reaching some remote communities due to transportation constraints and resource limitations, indicating that additional strategies may be needed to reach the most isolated populations.

The integration of multiple child health services during ICHDs proved to be an important feature for caregivers. Among ICHD attendees, while 79.4% received vaccination services, substantial proportions also received vitamin A supplementation (48.6%) and deworming services (51.1%). Focus group discussions revealed that this comprehensive service package was a key motivator for attendance. As one caregiver noted, ‘What motivates me is that we can get everything for our children in one visit’. However, we found no evidence that caregivers deliberately delayed vaccination visits to coincide with additional services, suggesting that the integrated approach enhanced service uptake without creating adverse timing effects. Instead, the combination of services appeared to strengthen the overall program attendance and acceptance, particularly in remote areas where regular healthcare access may be limited.

The evaluation revealed several challenges in implementing ICHDs, including inadequate social mobilization, caregiver confusion due to multiple vaccination campaigns, delayed and insufficient vaccine supplies and funds, and the perceived adverse treatment of caregivers by HCWs. These challenges align with those experienced in similar settings in the African region, hampering the achievement of immunization coverage targets [13]. The findings further justify the need for additional resources to facilitate adequate training for health workers in timely vaccine acquisition and delivery, interpersonal skills, and communication with caregivers to provide services in a more enabling environment [2,23].

This evaluation also identified concerns about the safety of routine vaccines as one reason caregivers did not utilize immunization services, which is aligned with a previous study in Uganda [24]. To enhance the effectiveness of the ICHD program in Uganda, a comprehensive approach focused on debunking myths and misconceptions about vaccination, including the safety of routine vaccines, is essential. This can be achieved through a well-structured mobilization strategy that leverages targeted messaging. These messages, crafted with precise and comprehensive information about ICHDs, should be disseminated via trusted community figures and healthcare professionals. VHTs and HCWs play a pivotal role and should receive adequate training to convey accurate information about vaccines, their potential side effects, and management strategies for adverse reactions.

Strengthening the ICHDs also necessitates improved strategic planning and coordination at district and national levels. This includes ensuring that budgets are allocated in a timely manner, not only for vaccine procurement and distribution but also for the prompt compensation of HCWs. Additionally, it is vital to manage the scheduling of ICHDs and other vaccination campaigns effectively. This scheduling should be planned to avoid overlap and confusion, enabling caregivers to understand the specific objectives of each campaign and the services offered. Moreover, incorporating community feedback mechanisms, utilizing mass media for broader reach, and engaging in school-based educational programs could further reinforce the campaign’s objectives. These strategies, combined with a focus on logistical efficiency and accessibility, especially in remote areas, are crucial to enhancing the overall impact of the ICHD program in Uganda.

### Limitations

While this study has certain methodological constraints, some findings highlight the program’s strengths in achieving its core objectives. Although we relied on vaccination records to reduce recall bias, with approximately 67% of the sampled children having vaccination records, this represents reasonable documentation rates for the setting. The observed pattern of delayed vaccination, rather than being solely a limitation, demonstrates the program’s success in its primary goal—serving as an effective catch-up strategy for children who missed their routine vaccination schedule. This aligns with the fundamental purpose of ICHDs, which is to periodically intensify routine immunization services to reach children who might otherwise remain unvaccinated.

In addition, the potential respondent selection bias in the household surveys, KIIs, and FGDs is another consideration. The lack of vaccination records in some cases required reliance on caregivers’ recollections, though this is a common challenge in vaccination coverage surveys in similar settings. Furthermore, interviewer or facilitator biases could have influenced the participant responses, potentially skewing the data. A mixed-methods approach was utilized to mitigate these biases, enabling data triangulation from different sources. The use of structured and piloted tools for the household surveys, KIIs, and FGDs, as well as purposive sampling, was intended to enhance the consistency and reliability of the data.

A notable limitation of the study is its restricted generalizability. The findings are specific to the three districts where the evaluation was conducted and may not apply to other regions in Uganda. Future studies could benefit from a more expansive, nationally representative sample for broader applicability. Additionally, the unavailability of reliable administrative immunization data posed a significant limitation, hindering comprehensive data triangulation. This constraint underlines the need for improved accessibility to reliable data in future evaluations to ensure a more holistic understanding of the ICHD program’s impact across Uganda. While these limitations present challenges, the study’s findings still offer important insights into implementing ICHDs in Uganda, guiding future improvements and research directions.

## 5. Conclusions

While the ICHDs in Uganda have faced systematic and operational challenges, they have notably enabled many children to access routine vaccination services. However, an important finding of this study is the juxtaposition of a high overall immunization rate against the finding of delayed vaccinations among those who have been immunized. This indicates that while ICHDs may have contributed toward increased vaccination access, there remains a gap in ensuring timely immunization for all children. It underscores the need for enhanced coordination and mobilization within the UNEPI to extend coverage and address delayed vaccinations. Such optimized efforts are crucial for achieving universal accessibility and effectively reaching all children with lifesaving vaccines in Uganda.

## Figures and Tables

**Table 1:** Characteristics of caregivers of children < 5 years of age based on household survey in Rakai, Kayunga, and Bukedea districts, Uganda, 2022 (n = 1432).

Variable	Frequency (N)	Percentage (95% CI)
Age of child		
0–9 months	561	40.7 (37.9–43.6)
10–18months	380	26.0 (23.5–28.6)
19–24months	130	8.6 (7.0–10.2)
25–36 months	154	10.9 (9.1–12.6)
37–59 months	207	13.8 (11.8–15.7)
**Relationship of caregiver with child (%)**		
Mother	1098	76.7 (77.1–81.0)
Father	74	5.2 (3.5–5.5)
Grandmother	199	13.9 (10.9–14.2)
Others (grandfather, older sibling, etc.)	61	4.2 (2.9–4.9)
**Place of birth for the child**		
Home-no skilled attendance	201	12.9 (10.3-15.5)
Health Facility	1283	79.9 (77.8–82.9)
Traditional Birth Attendant	78	6.3 (3.5-9.1)
Don’t Know	8	0.5 (0.1-1.0)
**Caregiver’s highest education (%)**		
No formal education/some primary school	904	63.1 (59.0–67.8)
Completed primary school or above	528	36.9 (32.8–40.3)
**Sources of income of caregiver (%)**		
Subsistence farming	1028	71.8 (70.2–74.7)
Does not work	135	9.4 (7.5–11.1)
Retail trade	96	6.7 (4.3–8.3)
Commercial farming	73	5.1 (2.3–7.6)
Formal employment	23	1.6 (0.2–2.7)
Commercial trade	13	0.9 (0.01–3.9)
Others	54	3.8 (1.6–6.3)
**Antenatal clinic attendance (mothers) (%)**	1221	97.4 (75.1–79.3)

The number of participants who answered each question differs.

**Table 2:** Vaccination status of children < 5 years of age based on household survey in Rakai, Kayunga, and Bukedea districts, Uganda, 2022 (n = 1432).

Variable	Frequency (N)	Percentage (95% CI)
**The child received all recommended vaccines for their age**	935/1376	69.4 (66.8–72.1)
**Presence of Child Health Card**	980/1376	67.1 (64.3–69.8)
**Vaccination Coverage based on Child Health Card for children aged 9–59 months (% received) (N = 793)**		
Bacillus Calmette–Guerin (BCG)	782	98.6 (97.6–99.7)
Penta ^1^ 1st dose	781	98.5 (97.5–99.4)
Penta 2nd dose	772	97.8 (96.8–98.8)
Penta 3rd dose	751	95.5 (94.0–96.9)
Oral polio vaccine (OPV) 1st dose	745	94.6 (92.9–96.3)
OPV 2nd dose	748	93.7 (91.7–95.8)
OPV 3rd dose	741	93.7 (91.8–95.6)
Pneumococcal Conjugate Vaccine (PCV) 1st dose	763	96.3 (94.9–97.8)
PCV 2nd dose	751	95.5 (93.9–97.1)
PCV 3rd dose	731	93.4 (91.6–95.3)
Rotavirus 1st dose	713	90.6 (88.4–92.8)
Rotavirus 2nd dose	695	88.3 (85.8–90.8)
Measles-rubella (MR) 1st dose	717	90.3 (88.1–92.6)
MR 2nd dose ^2^	140	16.2 (13.5–19.0)
**Zero Dose Children** No Penta recorded (0–59 months) (n = 980)	33	3.3 (2.0–4.3)
**Timeliness of vaccinations**		
Penta 1 (>8 weeks = delayed)	301	38.5 (34.8–42.1)
Penta 2 (>4 weeks after Penta1 = delayed)	489	54.7 (51.1–58.4)
Penta 3 (>4 weeks after Penta2 = delayed)	458	55.3 (51.5–59.0)
OPV 1 (>8 weeks = delayed = delayed)	294	39.3 (35.6–43.1)
OPV 2 (>4 weeks after OPV1 = delayed)	433	52.4 (48.6–56.2)
OPV 3 (>4 weeks after OPV2 = delayed)	461	57.4 (53.6–61.1)
PCV 1 (>8 weeks = delayed)	288	36.9 (33.3–40.5)
PCV2 (>8 weeks after PCV1 = delayed)	455	53.1 (49.4–56.8)
PCV3 (>8 weeks after PCV2 = delayed)	444	55.1 (51.3–58.9)
Rota 1 (>8 weeks = delayed)	324	44.1 (40.3–47.9)
Rota 2 (>8 weeks after RTO1 = delayed)	420	55.6 (51.7–59.5)
MR 1st dose (>10 months = delayed)	191	29.6 (25.7–33.5)
MR 2nd dose (<18 months)	47	34.9 (25.7–44.2)
MR 2nd dose (18–24 months)	24	16.6 (9.9–23.3)
MR 2nd dose (>24–35 months)	20	13.9 (7.7–20.1)
MR 2nd dose (36–59 months)	41	34.5 (25.0–44.1)

^1^ The Pentavalent vaccine protects children from diphtheria, pertussis, tetanus, hepatitis B, and *Hemophilus influenza* type B; ^2^ MR2 is due at 18 months of age, so children < 18 months of age were excluded from the denominator when calculating uptake of this vaccine.

**Table 3:** Caregivers’ knowledge, attitude, and practices related to Integrated Child Health Days in Uganda based on the household survey in Rakai, Kayunga, and Bukedea districts, Uganda, 2022.

Variable	Frequency (N)	Percentage (%) (95% CI)
**Knowledge of the existence of ICHDs (%)**	801/1077	74.7 (71.9–77.6)
**Reasons for attendance of ICHDs (%)**		
Immunization of children	585	91.9 (89.5–94.4)
Deworming	228	37.1 (32.9–41.4)
Growth monitoring	74	10.6 (8.0–13.2)
Others (infant HIV diagnosis, family planning, etc.)	99	15.3 (9.4–11.4)
**Site attended at most recent ICHDs (%)**		
Outreach	534	85.0 (81.7–85.5)
Health facilities	97	15.0 (11.9–18.0)
**Means used to attend most recent ICHDs (%)**		
Walking	511	80.4 (76.8–83.9)
Other means of transportation (Bicycle, Boda-Boda, car)	120	19.6 (17.0–20.5)
**Frequency of attending most recent ICHDs (%)**		
One-time visit	489	82 (78.9–85.1)
Two or more visits	107	18 (14.9–21.1)
**Distance traveling to ICHD site (%)**		
Less than 5 Km	496	85.6 (82.5–88.8)
**When the next ICHD will be held (%)**		
% who knew the correct time	132	20.9 (19.4–23.0)
**Source of information on ICHDs (%)**		
Health worker	160	18.3 (15.5–21.1)
Megaphone outreach	273	17.9 (17.4–20.5)
Village Health Team	745	49.0 (46.6–51.6)
Radios	174	11.4 (9.8–13.1)
WhatsApp groups	207	13.6 (13.1–15.6)
**Services received during ICHDs (%)**		
Immunization of children	503	79.4 (75.9–82.9)
Deworming	323	51.1 (46.7–55.4)
Vitamin A supplementation	321	48.6 (44.3–53.0)
Others (growth monitoring, HIV diagnosis, family planning)	140	10.9 (10.0–12.7)
**ICHDs services sought (%)**		
Immunization of children	536	84.3 (81.1–87.6)
Deworming of children	295	46.2 (41.8–50.3)
Vitamin A supplementation	252	37.5 (33.3–41.6)
Others (growth monitoring, nutrition education)	139	12.9 (10.5–14.3)

## Data Availability

The data can be made available upon request.

## Supplementary Materials

The following supporting information can be downloaded at: https://www.mdpi.com/article/10.3390/vaccines12121353/s1, The interview guides for KIIs and FGDs.

## Appendix A. Uganda National Expanded Program for Immunization Childhood Routine Immunization Schedule

1st	AT BIRTH	Polio 0	- Polio
		BCG	- Tuberculosis
2nd	AT 6 WEEKS (One and a half months)	Polio 1	- Polio
		DPT-Hep B-Hib 1	- Diphtheria - Whooping cough - Tetanus - Hepatitis B - Hemophilus influenza type B
		Pneumococcal Conjugate Vaccine 10 (PCV1)	- Meningitis - Pneumonia (caused by streptococcal pneumonia)
		Rotavirus Vaccine1	- Diarrhea
3rd	AT 10 WEEKS (Two and a half months)	Polio 2	- Polio
		DPT-Hep B-Hib 2	- Diphtheria - Whooping cough - Tetanus - Hepatitis B - Hemophilus influenza type B illnesses
		Pneumococcal Conjugate Vaccine 10 (PCV 2)	- Meningitis - Pneumonia (caused by streptococcal pneumonia)
		Rotavirus Vaccine 2	Diarrhea (caused by Rotavirus)
4th	AT 14 WEEKS (Three and a half months)	Polio 3	- Polio
		Injectable Polio Vaccine (IPV)	- Polio
		Pneumococcal Conjugate Vaccine 10 (PCV 3)	- Diphtheria - Whooping cough - Tetanus - Hepatitis B - Hemophilus influenza type B illnesses
	At 6 months and every 6 months until the child is 5 years	Vitamin A Supplement	- Prevent blindness and strengthen resistance to other diseases
5th	At 9 months	MR 1	- Measles-rubella
6th	At 18 months	MR 2 (The routine administration of the 2nd dose of the MR vaccine in Uganda started in October 2022.)	- Measles-rubella
Bacillus Calmette–Guerin (BCG); oral polio vaccine (Polio); diphtheria–tetanus–pertussis (DPT)–Hepatitis B (Hep B)–Hemophilus influenza type b (Hib); Pneumococcal Conjugate Vaccine (PCV); rotavirus vaccine (Rota)); measles-rubella vaccine (MR).			

## References

[1] WHO (2020). Immunization Agenda 2030: A Global Strategy to Leave No One Behind.

[2] Mihigo R., Okeibunor J., Anya B., Mkanda P., Zawaira F. (2017). Challenges of immunization in the African Region. Pan Afr. Med. J..

[3] Bangura J.B., Xiao S., Qiu D., Ouyang F., Chen L. (2020). Barriers to childhood immunization in sub-Saharan Africa: A systematic review. BMC Public Health.

[4] WHO (2009). Periodic Intensification of Routine Immunization: Lessons Learned and Implications for Action.

[5] Doherty T., Chopra M., Tomlinson M., Oliphant N., Nsibande D., Mason J. (2010). Moving from vertical to integrated child health programmes: Experiences from a multi-country assessment of the Child Health Days approach in Africa. Trop. Med. Int. Health.

[6] World Health Organization (2009). WHO Vaccine-Preventable Diseases: Monitoring System: 2009 Global Summary (No. WHO/IVB/2009). https://iris.who.int/bitstream/handle/10665/70149/WHO_IVB_2009_eng.pdf.

[7] MoH-Uganda (2012). Uganda Immunization Policy, December 2012, 5th Draft. https://extranet.who.int/countryplanningcycles/sites/default/files/planning_cycle_repository/uganda/epi_policy_-_final.pdf.

[8] Mast T.C., Kigozi G., Wabwire-Mangen F., Sewankambo N., Serwadda D., Gray R., Wawer M., Black R. (2006). Immunisation coverage among children born to HIV-infected women in Rakai district, Uganda: Effect of voluntary testing and counselling (VCT). AIDS Care.

[9] Fiedler J.L., Semakula R. (2014). An analysis of the costs of Uganda’s Child Days Plus: Do low costs reveal an efficient program or an underfinanced one?. Food Nutr. Bull..

[10] Okello G., Izudi J., Ampeire I., Nghania F., Dochez C., Hens N. (2022). Two decades of regional trends in vaccination completion and coverage among children aged 12-23 months: An analysis of the Uganda Demographic Health Survey data from 1995 to 2016. BMC Health Serv. Res..

[11] Ministry of Health, UNEPI (2021). Vaccination and Immunisation (UNEPI).

[12] Mupere E., Babikako H.M., Okaba-Kayom V., Mutyaba R.B., Mwisaka M.N., Tenywa E., Lule A., Aceng J.R., Mpanga-Kaggwa F., Matseketse D. (2020). Family Health Days program contributions in vaccination of unreached and under-immunized children during routine vaccinations in Uganda. PLoS ONE.

[13] WHO (2019). Progress and Challenges with Achieving Universal Immunization Coverage 2018 WHO/UNICEF Estimates of National Immunization Coverage (Data as of July 2019).

[14] Angeles G., Silverstein H., Ahsan K.Z., Kibria M.G., Rakib N.A., Escudero G., Singh K., Mpiima J., Simmons E., Weiss W. (2023). Estimating the effects of COVID-19 on essential health services utilization in Uganda and Bangladesh using data from routine health information systems. Front. Public Health.

[15] Oliphant N.P., Mason J.B., Doherty T., Chopra M., Mann P., Tomlinson M., Nsibande D., Mebrahtu S. (2010). The contribution of child health days to improving coverage of periodic interventions in six African countries. Food Nutr. Bull..

[16] Thompson S.K. (2012). Sampling.

[17] UBOS (2016). The National Population and Housing Census 2014—Main Report.

[18] World Health Organization (2018). World Health Organization Vaccination Coverage Cluster Surveys: Reference Manual (No. WHO/IVB/18.09). https://apps.who.int/iris/handle/10665/272820.

[19] Ministry of Health of Uganda (2023). Question and Answer Booklet on Routine Immunization.

[20] Babirye J.N., Engebretsen I.M.S., Makumbi F., Fadnes L.T., Wamani H., Tylleskar T., Nuwaha F. (2012). Timeliness of Childhood Vaccinations in Kampala Uganda: A Community-Based Cross-Sectional Study. PLoS ONE.

[21] Arambepola R., Yang Y., Hutchinson K., Mwansa F.D., Doherty J.A., Bwalya F., Ndubani P., Musukwa G., Moss W.J., Wesolowski A. (2021). Using geospatial models to map zero-dose children: Factors associated with zero-dose vaccination status before and after a mass measles and rubella vaccination campaign in Southern province, Zambia. BMJ Glob. Health.

[22] Vijayaraghavan M., Wallace A., Mirza I.R., Kamadjeu R., Nandy R., Durry E., Everard M. (2012). Economic evaluation of a Child Health Days strategy to deliver multiple maternal and child health interventions in Somalia. J. Infect. Dis..

[23] Hosseinpoor A.R., Bergen N., Schlotheuber A., Gacic-Dobo M., Hansen P.M., Senouci K., Boerma T., Barros A.J.D. (2016). State of inequality in diphtheria-tetanus-pertussis immunisation coverage in low-income and middle-income countries: A multicountry study of household health surveys. Lancet Glob. Health.

[24] Braka F., Asiimwe D., Soud F., Lewis R.F., Makumbi I., Gust D. (2012). A qualitative analysis of vaccine safety perceptions and concerns among caretakers in Uganda. Matern. Child Health J..

