# Supplementary material for: Evaluation of Integrated Child Health Days as a Catch-Up Strategy for Immunization in Three Districts in Uganda
Source: Vaccines (Basel). 2024 Nov 29;12(12):1353. doi: 10.3390/vaccines12121353 (PMC11680261; doi:10.3390/vaccines12121353)
Supplement: Supplementary file 1 [file vaccines-12-01353-s001.zip › vaccines-3267721-supplementary.pdf]

Focus Group Discussion Guide for Primary Caregivers of children under 5 years that attended ICHDs

Main Objective: To evaluate the effectiveness of integrated child health days in Uganda to reach children who are under-immunized and suggest recommendations for improvement of the ICHD service delivery program in Uganda in select three high, medium and low performing districts

Informed consent: Conduct the consenting process and obtain signed forms

Introduction: My name is ____________________________. I am working ………………………… district. The Ministry of Health (MoH) in collaboration with ICAP and CDC is conducting a survey to evaluate the effectiveness of integrated child health days in Uganda to reach children who are under-immunized in select three high, medium and low performing districts. The purpose of the survey is to understand the implementation of the ICHDs program and suggest recommendations for improvement of the ICHD service delivery program in Uganda.

On behalf of MoH, we kindly request for your participation in this survey by responding to a few questions. The information you provide will be kept strictly confidential. We will not use your full names during the discussions or write down or record your names or any personal information that could identify you. You are free to stop the discussion and leave at any time. You can also refuse to answer any specific questions if they make you uncomfortable. The discussion will take about one hour. At this time, do you agree to participate in the survey?

Signature of respondent……………………………………………..

Title of respondent……………………………………………............

Socio-demographics of the respondents

No	Indicators	Response
1.	No. of female participants	
2.	No. of male participants	
3.	Ages of the caregivers	
4.	Marital Status of the caregivers	
5.	Education level of the caregivers	
6.	General Occupation of the caregivers	

Indicators	Caregivers of Children under 5 years
Date of interview	
Name of District	
Venue	
Language of interview	
Time discussion started	
Time discussion ended	
FGD identifier	
Names of facilitators	

**Knowledge about ICHDS and its benefits**
1. What do you know about **ICHDS**?<br>**Probe:**<br>a. What are ICHDs?<br>b. Which month were ICHDs conducted?<br>c. Which months are ICHDs usually conducted?<br><br>2. How was your experience been in attending ICHDs?<br>**Probe:**<br>a. What were the **benefits** of attending **ICHDS**?<br>b. What were the challenges in attending ICHDs?<br>c. What **services** were offered by ICHDs?<br>d. What services were not provided that should be provided?
**Practices during implementation of ICHDS services**
3. How are ICHDS services delivered in this community?<br>**Probe:**<br>a. Where do you access the ICHD services?<br>b. Are these services accessible by you?<br>c. Who provides the services?<br>d. Have you received any sensitization on ICHDs?<br>e. If yes, what type of sensitization did you receive regarding ICHDs?
**Attitudes about ICHDS services**
4. What is your point of view about the implementation of ICHDS services in your community?<br>**Probe:**<br>a. What is the general **perception** of the community towards ICHDs activities?<br>b. What is good about the ICHDS services provided in their community?<br>c. What is the level of **awareness** of ICHD services in the community?<br>d. Why do some caregivers <u>**refuse**</u> to take their children for immunization services?<br>e. Why do some caregivers **take** their children for immunization services?
**Enablers/Opportunities/Motivators/Facilitators of ICHDS effective implementation**
5. What enables you as caregivers in this community to participate in seeking for ICHDs services for your child/children under 5 years of age?<br>**Probe:**

At an Individual level:

a. What motivates caregivers to attend ICHDs? (Such may include mobilization, desiring to have a healthier baby, compliance to national guidance etc.).

b. Do you (caregivers) receive any support from family members/partner to attend ICHDS? If YES, what kind of support?

At Community level:

a. Do you (caregivers) receive support from the community members regarding attending ICHDS? (This may include religious leaders who speak and participate in community mobilization on ICHDS, social networks such as women groups, VHTs, local and cultural leaders and other community structures etc.).

b. Are there community beliefs that positively impact on immunisation?

Healthcare system:

a. How does the healthcare system enable caregivers and their children below 5 years of age in this community to attend ICHDS on schedule? (Availability of ICHDS logistics, behaviour of healthcare workers, communication from health care workers).

b. Do caregivers of children under 5 years in this community get any support from the healthcare providers to enable them attend ICHDS when scheduled? (May include constant reminders by healthcare providers during other health care services, friendliness of healthcare workers with caregivers and their children, etc.)

Threats/Challenges/Barriers to attendance of ICHDS

6. What hinders caregivers of children below 5 years in this community from attending ICHDS?

Probe:

Individual Level Challenges: What individual level challenges prevent caregivers of children below 5 years in this community from attending ICHDS?

Community Level Challenges: What community level challenges prevent caregivers of children below 5 years from attending ICHDS?

Health system level challenges: What health system challenges hinder caregivers of children below 5 years from attending ICHDS? (Probe for availability of ICHDS logistics, ICHDS schedules, ICHDS implementation sites (Static/Outreach days etc.)

Recommendations for improving ICHDS services

7. How can ICHDS services in this community be proved?

Probe:

a. Which strategies can be used to improve attendance of ICHDs in this community?

THANK YOU SO MUCH FOR YOUR TIME

Focus Group Discussion Guide for Primary Caregivers of children under 5 years that did not attend ICHDs

Main Objective: To evaluate the effectiveness of integrated child health days in Uganda to reach children who are under-immunized and suggest recommendations for improvement of the ICHD service delivery program in Uganda in select three high, medium and low performing districts

Informed consent: Conduct the consenting process and obtain signed forms

Introduction: My name is ____________________________. I am working ………………………… district. The Ministry of Health (MoH) in collaboration with ICAP and CDC is conducting a survey to evaluate the effectiveness of integrated child health days in Uganda to reach children who are under-immunized in select three high, medium and low performing districts. The purpose of the survey is to understand the implementation of the ICHDs program and suggest recommendations for improvement of the ICHD service delivery program in Uganda.

On behalf of MoH, we kindly request for your participation in this survey by responding to a few questions. The information you provide will be kept strictly confidential. We will not use your full names during the discussions or write down or record your names or any personal information that could identify you. You are free to stop the discussion and leave at any time. You can also refuse to answer any specific questions if they make you uncomfortable. The discussion will take about one hour. At this time, do you agree to participate in the survey?

Signature of respondent………………………………………………..

Title of respondent…………………………………………………..........

Socio-demographics of the respondents

No	Indicators	Response
**1.**	No. of female participants	
**2.**	No. of male participants	
**3.**	Ages of the caregivers	
**4.**	Marital Status of the caregivers	
**5.**	Education level of the caregivers	
**6.**	General Occupation of the caregivers	

Indicators	Caregivers of Children under 5 years
Date of interview	
Name of District	

Venue	
Language of interview	
Time discussion started	
Time discussion ended	
FGD identifier	
Names of facilitators	

Knowledge about ICHDS and its benefits

1. What do you know about **ICHDS**?

Probe:

a. What are ICHDs?
b. Which month were ICHDs conducted?
c. Which months are ICHDs usually conducted?

2. What are some of reasons for non-attendance of ICHDs?

Probe:

a. What were your **reasons** for **not attending ICHDs**?
b. What information do you think was **missing** that could have motivated you to attend?
c. What could motivate you to attend ICHDs next time?
d. What steps do you think organizers should consider enabling non attendees to attend ICHDS services provided in the community?

Practices during implementation of ICHDS services

3. How are ICHDS services delivered in this community?

Probe:

a. Where do you access the ICHD services?
b. Are these services accessible by you?
c. Who provides the services?
d. Have you received any sensitization on ICHDs?
e. If yes, what type of sensitization did you receive regarding ICHDs?

Attitudes about ICHDS services

4. What is your point of view about the implementation of ICHDS services in your community?

Probe:

a. What is the general **perception** of the community towards ICHDs activities?
b. What is good about the ICHDS services provided in their community?
c. What is the level of **awareness** of ICHD services in the community?
d. Why do some caregivers **refuse** to take their children for immunization services?
e. Why do some caregivers **take** their children for immunization services?

**Enablers/Opportunities/Motivators/Facilitators of ICHDS effective implementation**
5. What would enable you as caregivers in this community to participate in seeking for ICHDs services for your child/children under 5 years of age?<br>**Probe:**<br>**At an Individual level:**<br>**a.** What would motivate you as a caregiver to attend ICHDs? (Such may include mobilization, desiring to have a healthier baby, compliance to national guidance etc.).<br>b. Do you (caregivers) receive any support from family members/partner to attend ICHDS? If YES, what kind of support?<br>**At Community level:**<br>a. Do you (caregivers) receive support from the community members regarding attending ICHDS? (This may include religious leaders who speak and participate in community mobilization on ICHDS, social networks such as women groups, VHTs, local and cultural leaders and other community structures etc.).<br>b. Are there community beliefs that positively impact on immunisation?<br><br>**Healthcare system:**<br>a. How does the healthcare system enable caregivers and their children below 5 years of age in this community to attend ICHDS on schedule? (Availability of ICHDS logistics, behaviour of healthcare workers, communication from health care workers).<br>b. Do caregivers of children under 5 years in this community get any support from the healthcare providers to enable them attend ICHDS when scheduled? (May include constant reminders by healthcare providers during other health care services, friendliness of healthcare workers with caregivers and their children, etc.)
**Threats/Challenges/Barriers to attendance of ICHDS**
6. What hinders caregivers of children below 5 years in this community from attending ICHDS?<br>**Probe:**<br>**Individual Level Challenges:** What individual level challenges prevent caregivers of children below 5 years in this community from attending ICHDS?<br>**Community Level Challenges:** What community level challenges prevent caregivers of children below 5 years from attending ICHDS?<br>**Health system level challenges**: What health system challenges hinder caregivers of children below 5 years from attending ICHDS? (Probe for availability of ICHDS logistics, ICHDS schedules, ICHDS implementation sites (Static/Outreach days etc.)
**Recommendations for improving ICHDS services**
7. How can ICHDS services in this community be improved?

Probe:

a. Which strategies can be used to improve attendance of ICHDs in this community?

THANK YOU SO MUCH FOR YOUR TIME

KEY INFORMANT INTERVIEW (KII) GUIDE

Key Informant Interview Guide for National level officers	
**RESPONDENT**	**National Level: 2 UNEPI and 1 UNICEF Focal persons**
**Main Objective**	To evaluate the effectiveness of integrated child health days in Uganda to reach children who are under-immunized and suggest recommendations for improvement of the ICHD service delivery program in Uganda.
**Informed consent**	**Introduction:** My name is ______________________________. I am working ………………………… district. The Ministry of Health (MoH) in collaboration with ICAP and CDC is conducting a survey to evaluate the effectiveness of integrated child health days in Uganda to reach children who are under-immunized in select three high, medium and low performing districts. The purpose of the survey is to understand the implementation of the ICHDs program and suggest recommendations for improvement of the ICHD service delivery program in Uganda.<br><br>On behalf of MoH, we kindly request for your participation in this survey by responding to a few questions. The information you provide will be kept strictly confidential. We will not use your full names during the discussions or write down or record your names or any personal information that could identify you. You are free to stop the discussion and leave at any time. You can also refuse to answer any specific questions if they make you uncomfortable. The discussion will take about one hour. At this time, do you agree to participate in the survey?<br><br>Conduct the consenting process and obtain signed forms.
	**<u>Program Management (Planning, Coordination and Funding)</u>**<br><br>1. What is the purpose of the ICHDs in October 2022?<br><br>2. Overall, what are the key roles of UNEPI in the planning, implementation and monitoring of ICHDs at national level?<br><br>**Planning and Coordination of ICHDs**<br>3. How are ICHDs planned for?<br>**Probe:**<br>-Who are the Stakeholder and key personnel involved?<br>-Which departments within MoH that are involved in planning and coordinating ICHDs?<br>-What sub-committees are involved in the planning?<br>-How long does planning take?<br>-What are key items for planning?<br>-What targets are set?<br>-What are the objectives of ICHDs?

	-How are ICHD guidelines/directives disseminated to the districts?<br><br>4. Describe **preparation and coordination** processes; national level supportive supervision during implementation.<br>**Probe**:<br>-Which key personnel are involved in coordination of ICHDs?<br>- Which key personnel are involved in supervision of ICHDs?<br><br>**Funds disbursement and accountability of ICHDs**<br>5. Describe the process of funds disbursement to the districts.<br>**Probe:**<br>-What is the source of funds for ICHDs?<br>-Are the funds readily available every April and October?<br>-How funds are received and disbursed at various levels of health system<br>-How much is sent out in total?<br>-Is this amount sufficient?<br>-Is the amount sent to districts consistent each year?<br>-When (month) are the funds sent out?<br>-Do districts receive these funds on time?<br>6. How are the funds accounted for?<br>**Probe:**<br>-Are there guidelines on use of funds/budget lines?<br>-What is the availability and use of accountability guidelines?<br>-Who is mandated to account?<br><br>-What is the time frame for submission of accountabilities to national level?<br>-When are districts supposed to submit financial reports<br>-What is timeliness of submission of financial reports to national level?<br><br>7. Describe the process of submission of activities' report.<br>**Probe:**<br>-What is the availability and use of reporting templates?<br>-When is the report supposed to be submitted?<br>What is the timeliness of submission of technical reports from districts to national level?<br><br>**<u>Cold Chain, logistics and Vaccine Management</u>**<br>8. Please describe the process of Supply chain management for vaccines and supplies for ICHDs at the national level and distribution to district level and then facility level.<br>**Probe**:<br>-Is the Evaluation of Cold Chain done prior to ICHD implementation?<br>-Where are vaccines stored at national level?<br>-How and when are vaccines distributed to all districts in time for ICHDs?<br>-Are vaccines and supplies sent out in time?<br>-Is cold chain equipment capacity sufficient at district level for vaccines during ICHDs?

	9. What challenges are associated with the above process of supply chain and vaccine management during ICHDs?<br>-Any solutions that have been used to overcome these challenges, and were they successful?<br><br>**<u>Service delivery</u>**<br>10. Please describe the packages of ICHDs and process of service delivery of the ICHD packages.<br>**Probe:**<br>-What packages were given in 2022?<br>-How have packages changed over time (in last 5 years)<br><br>11. Is there supportive supervision for ICHDs?<br>**Probe:**<br>-When, how, and who conducts supportive supervision from national level?<br>-From sub-national level?<br>-Are there national tools used for supportive supervision?<br><br>12. Please describe the potential of ICHDs in reaching zero dose/under-immunised children<br>**Probe:**<br>-In your opinion, how effective are ICHDs in reaching under-immunised and/or zero-dose children?<br>-What are strengths and weaknesses of ICHDs in reaching under-immunised /zero dose children<br>-Are there any known specific population subgroups with unimmunised children?<br>-Is there presence of vaccine hesitant populations<br>-Are there religious sects/cultural groups which are vaccine hesitant<br>-Are there underserved/hard to reach populations<br>-What strategies are used to reach the above populations?<br><br>**<u>Monitoring (records, data management, AEFIs)</u>**<br>13. Please describe process of data monitoring for ICHDs.<br>**Probe:**<br>-Are extra data collection tools (in addition to those for Routine Immunization) distributed to districts?<br>-How is national ICHD performance determined?<br>-Are there set targets for ICHD performance at national level? How are targets set?<br>-Are districts with sub-optimal performance followed up?<br>-How are AEFIs reported during ICHDs?<br><br>**<u>Advocacy, Communication and Social Mobilization</u>**<br>14. Please describe the processes for Advocacy, Communication and Social Mobilization for ICHDs?<br>**Probe:**<br>- Are there specific plans for Advocacy, Communication and Social Mobilization (ACSM)?

	- What platforms are used for ACSM?<br>- When is the timing of social mobilization?<br>- Are funds sufficient for social mobilization?<br>- Who is involved in ACSM?<br>- How long does social mobilization last during ICHDs?<br>- When does it start?<br>- How are hard to reach areas/populations mobilized?<br><br>**General Perspectives**<br>15. In your opinion, how important or useful are ICHDs to increase vaccination coverage beyond what is possible through routine immunization activities?<br>16. In your opinion, how did the COVID-19 pandemic impact the planning, coordination, and resources for ICHDs?<br><br>**Gaps/Challenges/barriers**<br>17. What capacity gaps for ICHDs service delivery (remain/persist/) at the national level.<br>**Probe:**<br>- Are the vaccines adequate?<br>- What are the Cold chain challenges at national or subnational level (storage, packaging, etc)<br>- What are the Human resource challenges (at different levels of health system)<br>- What are the transportation challenges?<br>- What are the challenges in availability of allowances for staff during ICHDs?<br>- What are challenges in capacity of VHTs to support immunization?<br>- Any challenges in Program management (Planning, funding, and accounting for ICHDs)?<br>- Any challenges in Advocacy, Communication and Social mobilization?<br>- Any challenges in M&E?<br><br>**Motivators/Opportunities**<br>18. What are some of the opportunities for increasing ICHDs performance in Uganda?<br>**Probe:**<br>-What are the potential areas (Funding, coordination, human resources, planning, monitoring, advocacy) for improving the reach of ICHDs?<br><br>**Recommendations**<br>19. In your view, what can be done to improve ICHDs delivery in Uganda?<br>**Probe:**<br>-What strategies can be implemented to improve ICHDs?<br><br>**Thank you so much for your time**

Key Informant Interview Guide for District officers	
**Respondent**	**DHT members District Health Officer (DHO) and Assistant District Health Officer in charge of Maternal and Child Health (ADHO-MCH)**
**Main objective**	To evaluate the effectiveness of integrated child health days in Uganda to reach children who are under-immunized and suggest recommendations for improvement of the ICHD service delivery program in Uganda.
**Informed Consent**	**Introduction:** My name is ______________________________. I am working ………………………… district. The Ministry of Health (MoH) in collaboration with ICAP and CDC is conducting a survey to evaluate the effectiveness of integrated child health days in Uganda to reach children who are under-immunized in select three high, medium and low performing districts. The purpose of the survey is to understand the implementation of the ICHDs program and suggest recommendations for improvement of the ICHD service delivery program in Uganda.<br><br>On behalf of MoH, we kindly request for your participation in this survey by responding to a few questions. The information you provide will be kept strictly confidential. We will not use your full names during the discussions or write down or record your names or any personal information that could identify you. You are free to stop the discussion and leave at any time. You can also refuse to answer any specific questions if they make you uncomfortable. The discussion will take about one hour. At this time, do you agree to participate in the survey?<br><br>Conduct the consenting process and obtain signed forms.
	**Program Management (Planning, Coordination and Funding)**<br>1. Overall, what are your key roles in the implementation of ICHDs in your district?<br>2. What is the purpose or main objectives of ICHDs in 2022?<br><br>**Planning and Coordination of ICHDs**<br>3. How are ICHDs planned and coordinated in this district?<br>**Probe:**<br>-Who are the Stakeholder and key personnel involved?<br>-What Departments within district that are involved in planning and coordinating ICHDs?<br>-What sub-committees are involved in the planning of ICHDs?<br>-How long does planning take?<br>-What are key items for planning?<br>-What targets are set during planning?<br>-What are the objectives of ICHDs?<br>-Which guidelines are followed and where are these guidelines from?<br>-What is the content of the guidelines?

	4. Describe **preparation and coordination** processes at district level supportive supervision during implementation.<br>**Probe**:<br>-Which key personnel are involved in coordination of ICHDs?<br>- Which key personnel are involved in supervision of ICHDs?<br><br>**Funds disbursement and accountability of ICHDs**<br><br>5. Describe the process of funds of receiving funds from the national level.<br>**Probe:**<br>-What is the source of funds for ICHDs?<br>-Are the funds readily available every April and October?<br>-How funds are received and disbursed at various levels of health system-from districts to all health facilities?<br>-Which facilities receive the funds?<br>-How much funds are received?<br>-How much are distributed?<br>-Is this amount sufficient?<br>-Is the amount sent to districts consistent each year?<br>-When (month) are the funds received?<br>-Does the district receive these funds on time?<br>6. How are the funds accounted for?<br><br>**Probe:**<br>-Are there guidelines on use of funds/budget lines?<br>-What is the availability and use of accountability guidelines?<br>-Who accounts for these funds?<br>-What is the time frame for submission of accountabilities to national level?<br>-When are districts supposed to submit financial reports?<br>-What is timeliness of submission of financial reports to national level?<br><br>7. Describe the process of submission of activities' report.<br>**Probe:**<br>-What is the availability and use of reporting templates?<br>-When is the financial and technical report supposed to be submitted?<br>-What is the timeliness of submission of financial and technical reports from districts to national level?<br><br>**<u>Cold Chain, logistics and Vaccine Management</u>**<br>8. Please describe the process of Supply chain management for vaccines and supplies for ICHDs from district level to all health facilities.<br>**Probe**:<br>-Is the Evaluation of Cold Chain done prior to ICHD implementation?

	-Where are vaccines received from NMS -How many months prior implementation of ICHDs??<br>-How and when are vaccines distributed to all health facilities in time for ICHDs?<br>-Are vaccines and supplies sent out in time?<br>-Is cold chain equipment capacity sufficient at district level for vaccines during ICHDs?<br><br>9. What challenges are associated with the above process of supply chain and vaccine management during ICHDs?<br>-Any solutions that have been used to overcome these challenges, and were they successful?<br><br>**<u>Service delivery</u>**<br>10. Please describe the packages of ICHDs and process of service delivery of the ICHD packages.<br>**Probe:**<br>-What packages were given in 2022?<br>-How have packages changed over time (in last 5 years)<br><br>11. Is there supportive supervision for ICHDs?<br>**Probe:**<br>-When, how and who conducts supportive supervision from district and sub-district level?<br>-Are there tools used for supportive supervision?<br><br>12. Please describe the potential of ICHDs in reaching zero dose/under-immunised children.<br>**Probe:**<br>-In your opinion, how effective are ICHDs in reaching under-immunised and/or zero-dose children?<br>-What are strengths and weaknesses of ICHDs in reaching under-immunised /zero dose children<br>-Are there any known specific population subgroups with unimmunised children?<br>-Is there presence of vaccine hesitant populations<br>-Are there religious sects/cultural groups which are vaccine hesitant<br>-Are there underserved/hard to reach populations<br><br>-What strategies are used to reach the above populations?<br><br>**<u>Monitoring (records, data management, AEFIs)</u>**<br>13. Please describe process of data monitoring for ICHDs.<br>**Probe:**<br><br>-Are extra data collection tools (in addition to those for Routine Immunization) received from NMS?

	-Are extra data collection tools distributed to the health facilities?<br>-How is district ICHD performance determined?<br>-Are there set targets for ICHD performance at district?<br>-Are these targets communicated from national level?<br>-Are health facilities with sub-optimal performance followed up?<br>-How are AEFIs reported during ICHDs?<br><br>**<u>Advocacy, Communication and Social Mobilization</u>**<br>14. Please describe the processes for Advocacy, Communication and Social Mobilization for ICHDs?<br>**Probe:**<br>- Are there specific plans for Advocacy, Communication and Social Mobilization (ACSM)?<br>- What platforms are used for ACSM?<br>- When is the timing of social mobilization?<br>- Are funds sufficient for social mobilization?<br>- Who is involved in ACSM?<br>- How long does social mobilization last during ICHDs?<br>- When does it start?<br>- How are hard to reach areas/populations mobilized?<br><br>**General Perspectives**<br>15. In your opinion, how important or useful are ICHDs to increase vaccination coverage beyond what is possible through routine immunization activities.<br>**Gaps/Challenges/barriers**<br>16. What capacity gaps for ICHDs service delivery (remain/persist/) at the district level.<br>**Probe:**<br>- Are the vaccines adequate?<br>- What are challenges in service delivery-at static? At outreaches?<br>- What are the Cold chain challenges at district and health facility levels (storage, packaging, etc)<br>- What are the Human resource challenges (at district and health facility levels)<br>- What are the transportation challenges?<br>- What are the challenges in availability of allowances for staff during ICHDs?<br>- What are challenges in capacity of VHTs to support immunization?<br>- Any challenges in Program management (Planning, funding, and accounting for ICHDs)?<br>- Any challenges in Advocacy, Communication and Social mobilization?<br>- Any challenges in M&E?

	**Motivators/Opportunities**<br>17. What are some of the opportunities for increasing ICHDs performance in your district?<br>**Probe:**<br>-What are the potential areas (Funding, coordination, human resources, planning, service delivery, monitoring, advocacy) for improving the reach of ICHDs?<br><br>**Recommendations**<br>18. In your view, what can be done to improve ICHDs delivery in this District?<br>**Probe:**<br>-What strategies can be implemented to improve ICHDs?<br><br>**Thank you so much for your time**
**Key Informant Interview Guide for Health Workers**	
**RESPONDENT**	**In-charge health facility/in-charge immunization services at health facility**
**Main Objective**	To evaluate the effectiveness of integrated child health days in Uganda to reach children who are under-immunized and suggest recommendations for improvement of the ICHD service delivery program in Uganda.
**Informed Consent**	**Introduction:** My name is ______________________________. I am working ………………………….. district. The Ministry of Health (MoH) in collaboration with ICAP and CDC is conducting a survey to evaluate the effectiveness of integrated child health days in Uganda to reach children who are under-immunized in select three high, medium and low performing districts. The purpose of the survey is to understand the implementation of the ICHDs program and suggest recommendations for improvement of the ICHD service delivery program in Uganda.<br><br>On behalf of MoH, we kindly request for your participation in this survey by responding to a few questions. The information you provide will be kept strictly confidential. We will not use your full names during the discussions or write down or record your names or any personal information that could identify you. You are free to stop the discussion and leave at any time. You can also refuse to answer any specific questions if they make you uncomfortable. The discussion will take about one hour. At this time, do you agree to participate in the survey?<br><br>Conduct the consenting process and obtain signed forms.
	**<u>Program Management (Planning, Coordination and Funding)</u>**

	1. Overall, what are your key roles in the implementation of ICHDs) at this health facility?<br>2. What is the purpose of ICHDs in 2022?<br><br>**Planning and Coordination of ICHDs**<br>3. How are ICHDs planned and coordinated at this health facility?<br>**Probe:**<br>-Who are the Stakeholder and key personnel involved?<br>-What Departments within the health facility are involved in planning and coordinating ICHDs?<br>-What sub-committees are involved in the planning of ICHDs?<br>-How long does planning take?<br>-What are key items for planning?<br>-What targets are set during planning?<br>-What are the objectives of ICHDs?<br>-Which guidelines are followed and where are these guidelines from?<br>-What is the content of the guidelines?<br><br>4. Describe **preparation and coordination** processes at district level supportive supervision during implementation.<br>**Probe**:<br>-Which key personnel are involved in coordination of ICHDs?<br>- Which key personnel are involved in supervision of ICHDs?<br><br>**Funds disbursement and accountability of ICHDs**<br><br>5. Describe the process of funds of receiving funds from the district?<br>**Probe:**<br>-What is the source of funds for ICHDs?<br>-Are the funds readily available every April and October?<br>-How funds are received and disbursed at from district to last recipient?<br>-Who are the recipients of these funds?<br>-How much funds are received?<br>-How much are distributed?<br>-Is this amount sufficient?<br>-Is the amount sent from districts consistent each year?<br>-When (month) are the funds received?<br>-Does the health facility receive these funds on time?<br>6. How are the funds accounted for?<br>**Probe:**<br>-Are there guidelines on use of funds/budget lines?<br>-What is the availability and use of accountability guidelines?<br>-Who accounts for these funds?<br>-What is the time frame for submission of accountabilities to district level?

	-When are districts supposed to submit financial reports?<br>-What is timeliness of submission of financial reports to national level?<br><br>7. Describe the process of submission of activities' report.<br>**Probe:**<br>-What is the availability and use of reporting templates?<br>-When are the financial and technical reports supposed to be submitted?<br>-What is the timeliness of submission of financial and technical reports from districts to national level?<br><br>**<u>Cold Chain, logistics and Vaccine Management</u>**<br><br>8. Please describe the process of Supply chain management for vaccines and supplies for ICHDs from district level to all health facilities.<br>**Probe**:<br>-Is there evaluation of Cold Chain prior to ICHD implementation?<br>-When are vaccines received from NMS -How many months prior implementation of ICHDs?<br>-Are vaccines received in time for ICHDs?<br>-Is cold chain equipment capacity sufficient at health facility level for vaccines during ICHDs?<br><br>9. What challenges are associated with the above process of supply chain and vaccine management during ICHDs?<br>-Any solutions that have been used to overcome these challenges, and were they successful?<br><br>**<u>Service delivery</u>**<br>10. Please describe the packages of ICHDs and process of service delivery of the ICHD packages.<br>**Probe:**<br>-What packages were given in 2022?<br>-How have packages changed over time (in last 5 years)?<br>-How are these packages delivered to the end user?<br><br>11. Is there supportive supervision for ICHDs?<br>**Probe:**<br>-When, how and who conducts supportive supervision from at health facility level?<br>-Are there tools used for supportive supervision?<br><br>12. Please describe the potential of ICHDs in reaching zero dose/under-immunised children.<br>**Probe:**

	-In your opinion, how effective are ICHDs in reaching under-immunised and/or zero-dose children?<br>-What are strengths and weaknesses of ICHDs in reaching under-immunised /zero dose children<br>-Are there any known specific population subgroups with unimmunised children?<br>-Is there presence of vaccine hesitant populations?<br>-Are there religious sects/cultural groups which are vaccine hesitant?<br>-Are there underserved/hard to reach populations<br>-What strategies are used to reach the above populations?<br><br>**<u>Monitoring (records, data management, AEFIs)</u>**<br>13. Please describe process of data monitoring for ICHDs<br>**Probe:**<br>-Are extra data collection tools (in addition to those for Routine Immunization) given to this health facility?<br>-How is health facility ICHD performance determined?<br>-Are there set targets for ICHD performance at the health facility?<br>-Are these targets communicated from the district?<br>-Are outreaches with sub-optimal performance followed up?<br>-How are AEFIs reported during ICHDs?<br><br>**<u>Advocacy, Communication and Social Mobilization</u>**<br><br>14. Please describe the processes for Advocacy, Communication and Social Mobilization for ICHDs?<br>**Probe:**<br>- Are there specific plans for Advocacy, Communication and Social Mobilization (ACSM)?<br>- What platforms are used for ACSM?<br>- When is the timing of social mobilization?<br>- Are funds sufficient for social mobilization?<br>- Who is involved in ACSM?<br>- How long does social mobilization last during ICHDs?<br>- When does it start?<br>- How are hard to reach areas/populations mobilized?<br><br>**General Perspectives**<br>15. In your opinion, how important or useful are ICHDs to increase vaccination coverage beyond what is possible through routine immunization activities?<br><br>**Gaps/Challenges/barriers**<br>16. What capacity gaps for ICHDs service delivery (remain/persist/) at the health facility level?

	**Probe:**<br>- Are the vaccines adequate?<br>- What are challenges in service delivery-at static? At outreaches?<br>- What are the Cold chain challenges at health facility levels (storage, packaging, etc)<br>- What are the Human resource challenges (at district and health facility levels)<br>- What are the transportation challenges?<br>- What are the challenges in availability of allowances for staff during ICHDs?<br>- What are challenges in capacity of VHTs to support immunization?<br>- Any challenges in Program management (Planning, funding, and accounting for ICHDs)?<br>- Any challenges in Advocacy, Communication and Social mobilization?<br>- Any challenges in M&E?<br><br>**Motivators/Opportunities**<br>17. What are some of the opportunities for increasing ICHDs performance at this health facility?<br>**Probe:**<br>-What are the potential areas (Funding, coordination, human resources, planning, service delivery, monitoring, advocacy) for improving the reach of ICHDs?<br><br>**Recommendations**<br>18. In your view, what can be done to improve ICHDs delivery at this health facility?<br>**Probe:**<br>-What strategies can be implemented to improve ICHDs?<br><br>**Thank you so much for your time**

**Key Informant Interview Guide for Community Health Workers**	
**KII Respondents**	**Community Health Workers (CHWs)/Village Health Teams (VHTs)**
**Main Objective**	To evaluate the effectiveness of integrated child health days in Uganda to reach children who are under-immunized and suggest recommendations for improvement of the ICHD service delivery program in Uganda.
**Informed Consent**	**Introduction:** My name is ______________________________. I am working …………………………. district. The Ministry of Health (MoH) in collaboration with ICAP and CDC is conducting a survey to evaluate the effectiveness of integrated child health days in Uganda to reach children who are under-immunized in select three high, medium and low performing

	districts. The purpose of the survey is to understand the implementation of the ICHDs program and suggest recommendations for improvement of the ICHD service delivery program in Uganda.<br><br>On behalf of MoH, we kindly request for your participation in this survey by responding to a few questions. The information you provide will be kept strictly confidential. We will not use your full names during the discussions or write down or record your names or any personal information that could identify you. You are free to stop the discussion and leave at any time. You can also refuse to answer any specific questions if they make you uncomfortable. The discussion will take about one hour. At this time, do you agree to participate in the survey?<br><br>Conduct the consenting process and obtain signed forms.		
**Date**			
**KII characteristics**	Males:	Females:	
	Village:	Parish:	S/county:
	Health Facility attached to:	Note taker:	
**Roles and capacity of structures**	1. Do you assist during ICHDs?<br>2. Do you assist during Routine Immunization?<br>3. Describe the roles you play in the ICHDs services in your catchment area?<br>**Probe**:<br>a) Do you assist with community sensitization and mobilization?<br>b) Do you assist with vaccination of children (where- static, outreaches, hard to reach areas, what role- identifying defaulters or those due, filling register, checking card etc.)?<br>c) Do you assist with distribution of Vitamin A capsules and deworming tablets?<br>d) Do you assist with coordination of immunization outreaches?<br>e) How are you involved in assisting with sexual and reproductive health services?<br>f) How are you involved with assisting with nutrition, education and growth monitoring services?<br>4. Have you ever received any training in the following areas?<br>a) Immunization<br>b) Vitamin A supplementation<br>c) Deworming<br>d) Coordinating immunization outreaches,<br>e) Sexual and reproductive health services<br>f) Nutrition education and growth monitoring<br><br>5. Where there any refresher trainings offered in advance of ICHDs?<br>6. Which organization provided the training? For how long was the training?<br>7. What challenges do you face in performing your roles, particular those related to delivery of ICHDs?<br>8. In your opinion, how can these challenges be addressed?		
**Community perception of ICHDs activities**	9. In your opinion, how are ICHDs (immunization, Vitamin A supplementation and deworming, etc.) services useful in reaching under-immunized children beyond routine Immunization?		

	10. What is the general perception of the community towards ICHDs activities?<br>11. Is the community aware of ICHD services every April and October?<br>12. Are there community members who refuse to immunize their children in your area?<br>**Probe:**<br>a) Are you aware of vaccine hesitant populations in this community?<br>b) Are there a specific group of people who are vaccine hesitant? Which groups?<br>13. What could be some of the reasons for rejecting immunization, among these communities?<br>14. In your view, are there any children who have not got vaccinations in your catchment area and what are some reasons for non-vaccination?<br>**Probe:**<br>a) Is there presence of under-immunised children in your community?<br>b) Are you aware of children in your community who are eligible for immunization, but have never been vaccinated (zero-dose)?<br>c) What can be done to improve the uptake of ICHDs activities (immunization, Vitamin A supplementation and deworming services etc.) among this population?
**Challenges in the delivery of ICHDs**	15. What challenges do you face while supporting ICHDs (immunization, Vitamin A supplementation and deworming services) in your catchment area?<br>**Probe:**<br>a) Is there adequacy of vaccines during ICHDs?<br>b) Are there cold chain challenges (storage, packaging, etc)?<br>c) Are there Transportation challenges?<br>d) Is there difficulty in accessing hard to reach areas?<br>e) Are there challenges with allowances for VHTs?<br>f) Are there challenges in capacity of CHWs to support immunization (numbers & skills)<br>16. Are there communities which find it difficult to access ICHDs activities (immunization, Vitamin A supplementation and deworming services)?<br>**Probe:**<br>a) Hard to reach areas<br>b) Communities with no access to a health facility<br>17. What strategies can be used to reach such communities with immunization, Vitamin A supplementation and deworming services?<br>18. What can be done to improve the delivery of ICHDs activities in the community?<br>19. What strategies can improve communication and social mobilization?
**THANK YOU SO MUCH FOR YOUR TIME**	
